# Commentary: Embedding reflective practice into student nurse education about leadership and management theory

**DOI:** 10.1177/17449871251362921

**Published:** 2025-10-05

**Authors:** Elizabeth Jestico

**Affiliations:** Senior Lecturer in Children’s Nursing, Oxford Brookes University, Oxford, UK

Commentary on: Senior nursing students’ perspectives on nursing management: a qualitative study (Aydogdu and Disbudak, 2025).

This study by Aydogdu and Disbudak (2025) undertaken in Turkey about nursing students’ perspectives on nursing management and leadership in clinical practice was fascinating to read. I am the module leader for a United Kingdom (UK) pre-registration children’s nursing module that relates to nursing management and leadership, so this gave me much food for thought.

As the authors assert, whilst nursing management and leadership are important and mandatory components of nurse education worldwide, implementing effective education strategies in this area can be really challenging. In the UK, nursing curricula are developed in accordance with the Nursing and Midwifery Council’s standards of proficiency for registered nurses (2018). Leading and managing nursing care is one of the seven platforms within these standards and as such is a fundamental part of students’ learning in both theory and practice.

At the start of each module run, I am often greeted with students’ suspicious faces. They question why they need to be learning about leadership and change management theories which seem to them to lie in the remit of senior nurses holding management positions. These responsibilities are, in their opinion, far away from their roles as students or newly qualified nurses. My job as module leader is to ‘sell’ the importance of this to each cohort and empower them to take an active leadership role in their current career stage. This helps to break the assumption that nurses have to hold management roles to lead and simultaneously decouples the concepts of leadership and management. By the end of the module, having applied leadership theory to a quality improvement project that they propose, students recognise that leadership roles can be non-hierarchical. They reflect that as students and newly qualified nurses they do hold the potential to lead others in order to improve care for children and families.

However, this study highlights another important challenge. Whilst in the university setting, we may facilitate learning and engagement with leadership and management theories, this becomes ineffective if learning is not supported in practice. The authors of this study found that students’ observations of nurse managers in clinical practice influenced their perspectives of what good leadership looks like. This suggests that students who are not observing positive attributes or theories of leadership being displayed in practice, may find that their theoretical learning about leadership is impeded.

As nurse educators, we are often concerned about the ‘theory–practice gap’ (Maben et al., 2006). This study reinforces that this can apply to learning across the breadth of the curriculum. We have a responsibility to consider strategies to overcome this gap. Several years ago, I undertook a study exploring children’s nurses’ preparedness to care for children with cancer following their pre-registration education (Jestico and Finlay, 2017). A key finding of this was that integrating theory and practice during pre-registration education is fundamental to nurses’ confidence and competence once qualified. Participants expressed that learning in a purely theoretical way was unlikely to be effective, and it was experience in practice that would aid consolidation of their learning. It seems that there is one missing piece of the puzzle though. Whilst we can teach theory in university and students can gain experience in practice, they still need to be able to tie this theory and practice back together. Reflective practice enables the forging of these links between practice and theory learning, and by promoting reflection before, during and after practice experiences, we will best enhance students’ learning and thus their future practice (Patel and Metersky, 2022). The research study discussed here concluded that nursing students’ reflections can be drawn on to enhance curricula about management and leadership. These conclusions have led me to really consider how to narrow the theory/practice gap in these areas, and that further embedding reflective practice throughout the module that I run could enhance students’ learning and truly empower them to become nurse leaders of the future.

## References

[bibr1-17449871251362921] Aydogdu and Disbudak (2025) Senior nursing students’ perspectives on nursing management: a qualitative study. Journal of Research in Nursing 30: 622–640. DOI: 10.1177/17449871251370631 PMC12500597 41064154

[bibr2-17449871251362921] Jestico E Finlay T (2017) *“A* stressful and frightening experience”? Children’s nurses’ perceived readiness to care for children with cancer following pre-registration nurse education: A qualitative study. Nurse Education Today 48: 62–66. DOI: 10.1016/j.nedt.2016.09.019. 27710826

[bibr3-17449871251362921] Maben J Latter Sand Clark JM (2006) The theory–practice gap: impact of professional–bureaucratic work conflict on newly-qualified nurses. Journal of Advanced Nursing 55: 465–477. DOI: 10.1111/j.1365-2648.2006.03939.x 16866842

[bibr4-17449871251362921] Nursing and Midwifery Council (2018) Future Nurse: Standards of Proficiency for Registered Nurses. London: Nursing and Midwifery Council. Available at: https://www.nmc.org.uk/globalassets/sitedocuments/education-standards/future-nurse-proficiencies.pdf 10.1016/j.nedt.2024.106284 38870582

[bibr5-17449871251362921] Patel KM Metersky K (2022) Reflective practice in nursing: A concept analysis. International Journal of Nursing Knowledge 2022; 33(3): 180–187. DOI: 10.1111/2047-3095.12350. 34626459

